# Commentary: Rectangularity is stronger than symmetry in interpreting 2D pictures as 3D objects

**DOI:** 10.3389/fnhum.2023.1138287

**Published:** 2023-03-24

**Authors:** Tadamasa Sawada, Maria Dvoeglazova

**Affiliations:** ^1^European University of Armenia, Yerevan, Armenia; ^2^School of Psychology, HSE University, Moscow, Russia; ^3^Department of Psychology, Russian-Armenian (Slavonic) University, Yerevan, Armenia; ^4^Akian College of Science and Engineering, American University of Armenia, Yerevan, Armenia

**Keywords:** 3D mirror-symmetry, 3D rotational-symmetry, rectangularity, depth perception, visual illusion, shape constancy

## 1. Introduction

In this note, we are commenting on a paper by Sugihara and Pinna (2022, referred to as SP22 in this note) published in Frontiers in Human Neuroscience. SP22 discussed the role of rectangularity in the perception of 3D shapes based on the results of two behavioral experiments and on their observations of some visual illusions involving 3D shapes. We point out and explain several flaws in SP22.

## 2. Experiments of SP22

In SP22's experiments, SP22 examined the perceived shape of an object that was composed of two or three parts. Their observer responded by indicating whether the shape of the entire object was 3D mirror-symmetrical and whether the shapes of the parts were rectangular.

The experiments were not specifically designed to compare the roles of rectangularity and mirror-symmetry on the 3D shape perception. First, their observer's response about rectangularity was about local parts of the object while their response about mirror-symmetry concerned the entire object. These two types of response differed in both scale and type of regularity. Second, the two types of response needed two different response criteria. Note that, mathematically, mirror-symmetry is a discrete property but our visual system sees it as a continuous property (Sawada, 2010). So, the response about symmetry is based on both the perceived degree of symmetry and the criterion about symmetry. This can be also true for rectangularity.^1^ If it is, the responses about symmetry and rectangularity can be affected, individually, by their criteria used.

The visual stimuli used in SP22's experiments are images of only two 3D shapes. The effect of mirror-symmetry on the 3D shape perception can change, depending on some other properties of the shape, such as compactness (Sawada, 2010; Pizlo et al., 2014). The title of SP22 says “rectangularity is stronger than symmetry.” Such a general conclusion however, cannot be made solely on the basis of their two shapes.

**Figure 1:**
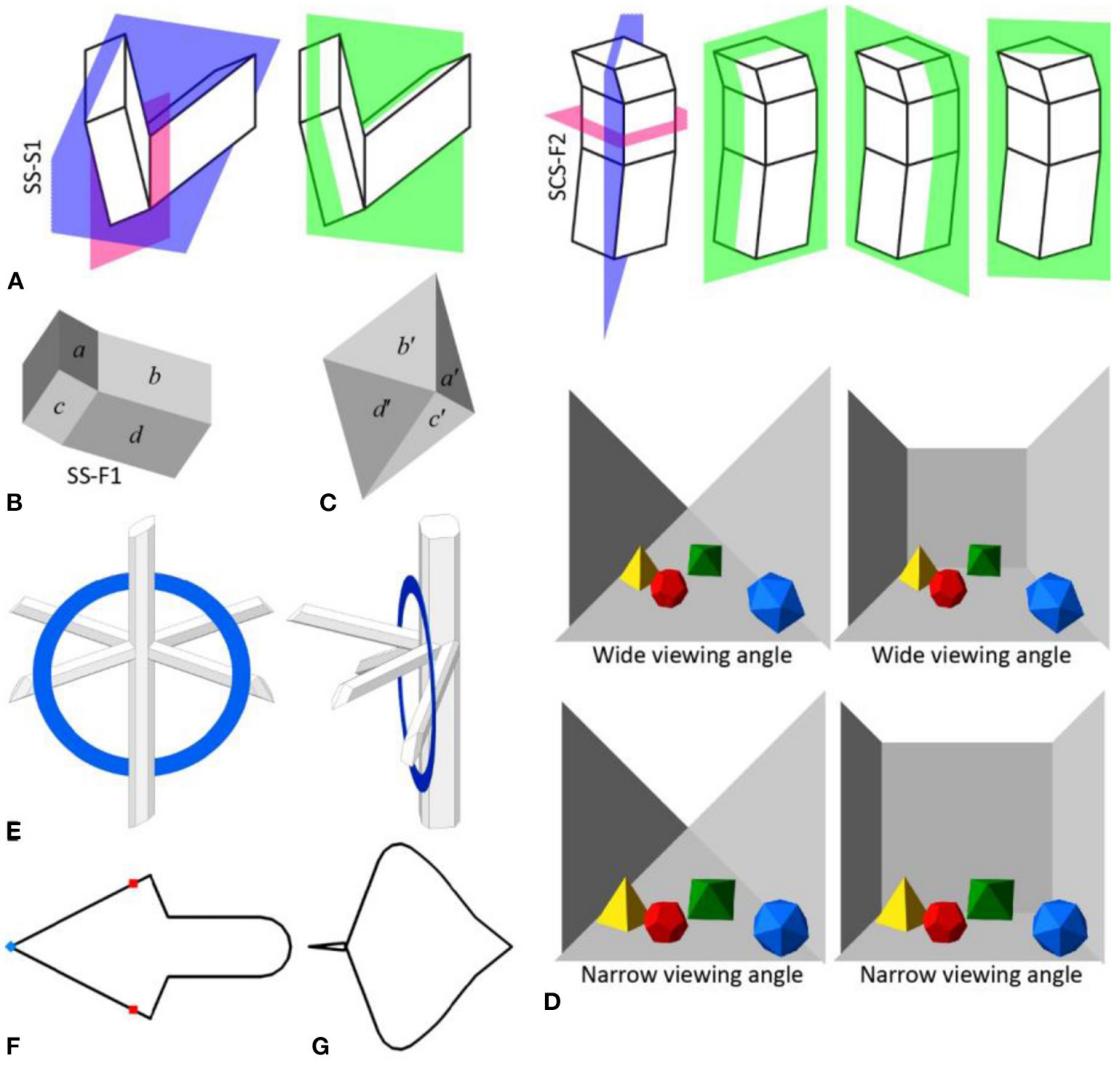
**(A)** Two of the visual stimuli used in SP22 (Sugihara and Pinna, 2022, SS-S1 in Figure 4A and SCS-F2 in Figure 6B) and their 3D mirror-symmetrical interpretations. Symmetry planes of their simulated 3D shapes that were considered by SP22 are in pink and those that were not considered are in blue. The symmetry-planes of other 3D mirror-symmetrical interpretations are in green. The additional symmetrical interpretations and their number change depending on the viewing direction of the shading image because of their topological inconsistency against the image (see Sawada and Pizlo, 2008). **(B)** A shading image of a 3D mirror-symmetrical shape used as a visual stimulus in SP22 (Sugihara and Pinna, 2022, SS-F1 in Figure 4B). Faces *a* and *c* are 3D mirror-symmetrical with faces *b* and *d*, respectively. Faces *b* and *c* are brighter than faces *a* and *d*. This pattern of shading cannot be generated with the simulated 3D mirror-symmetrical shape unless the pattern of reflectance is asymmetrical. Note that Lambertian reflectance and a single linear light source are assumed. **(C)** An image of a cone with a rhombus-shaped base. Faces *a*′, *b*′, *c*′, and *d*′ of the cone have the same orientations as faces *a, b, c*, and *d* of the simulated 3D shape in **(B)**. Assuming Lambertian and specular reflections, the faces of the cone would have the same pattern of reflection as the faces of the simulated shape under an orthographic projection. The cone is simply convex and it is easy to discuss its reflection pattern. According to **(B)**, the two opposite faces (*b*′ and *c*′) of the cone are brighter than the other two faces (*a*′ and *d*′). Such a diagonal pattern of reflection of the cone, which is simply convex, is at least as difficult unless reflectance is different between *a*′ and *b*′ or between *c*′ and *d*′. This reflectance pattern of the cone represents an asymmetrical pattern of reflectance of the simulated shape. **(D)** Perspective images of a room with and without a frontoparallel wall from a close distance with a wide viewing angle and from a far distance with a narrow viewing angle. The perceived depth of the polyhedral objects and of the fronto-parallel wall changes depending on the viewing-angle (dolly zoom, vertigo effect). The perceived shape of the room also changes depending on the viewing-angle when the fronto-parallel wall is shown. It is worth pointing out that the perceived 3D shapes of the polyhedral objects do not seem to change between the viewing angles but the shape of the room changes. **(E)** An impossible pair of objects made after Sugihara's (2005, 2014) “Four Perches and a Ring” without using any rectangular shapes (see https://tadamasasawada.com/demos/sp22/sugihara_4perches.html for an interactive demo). One of the objects is a planar ring. The other object is composed of octagonal and hexagonal cylinders. The end-faces of these cylinders are not normal to the axes of the cylinders. The cylinders and the composition of the cylinders do not have any rectangular trihedral corners. **(F)** A partial reproduction of Sugihara's (2016) “Right-Facing Arrow” (see https://tadamasasawada.com/demos/sp22/sugihara_arrow.html for an interactive demo). The original work is a cylinder with an open-top. Only the boundary contour of the open-top was reproduced. The contour is 3D rotationally-symmetrical. There are two types of degenerate views of the contour in this view of the contour. Some sharp-angles of the contour are not visible in the view (red dots, see Dickinson et al., 1999) and a sharp-angle in the view (a blue dot, see Koenderink, 1990; Sawada et al., 2014) is a projection of a smoothly-curved segment of the contour. **(G)** A different view of the contour in **(F)**. The contour is rotated by 90° around the symmetry-axis of the contour from the original view **(F)**. Images of the contour in this view and of the original view are 2D mirror-symmetrical. So, some other views that are close to these two views are approximately 2D skew-mirror-symmetrical.

The stimuli are not well controlled. Each simulated 3D shape of the stimulus was generated with the intention of having only one symmetry-plane but it also has a different symmetry-plane. Most of SP22's stimuli actually have a few other 3D interpretations that are mirror-symmetrical in different ways (Figure 1A). Such additional interpretations are different across the images of the same 3D shape. SP22 discussed whether the shape perceived is mirror-symmetrical in the same way that SP22 considered it to be. But, the perceived shape could be mirror-symmetrical in a way that had not been considered.

The perceived pattern of surface reflectance of a perceived shape is likely asymmetric even when the perceived shape is mirror-symmetrical (Figures 1B, C). The detection of 2D mirror-symmetry of a planar shape is disrupted by an asymmetric luminance pattern (Wenderoth, 1996). It is possible that the asymmetric reflectance pattern of the shape also disrupted the detection of the 3D mirror-symmetry of the shape.

## 3. Illusions of SP22

The 3D shapes used in the illusions discussed in SP22 are either rectangular or are perceived to be rectangular. There are some other factors in the illusions, however, that SP22 did not consider and they can also explain the illusions. The illusions can be reproduced without using any rectangular shapes (Figures 1D–F, see Supplementary material). This means that rectangularity is not essential for explaining the illusions. Then, it is at least difficult based on the illusions to discuss the role of rectangularity.

The first illusion is about the perceived shapes of a room from its photos taken from different viewing-angles (Figures 9A, B in SP22). The perceived shape changes depending on the viewing-angles. This illusion can be explained by linear-perspective (Figure 1D; Howard, 2012). The position of the fronto-parallel wall of the room changes depending on the viewing-angle but the horizontal edges of the side walls remain perceived as parallel to the depth axis. This changes the rooms' shape while the trihedral corners of the room remain rectangular. So, the illusion can be explained without rectangularity.

The photos of the first illusion have infinitely many 3D interpretations including square-frustums but the frustums are not perceived from the photos. SP22 thought that it was because the frustums do not have any rectangular trihedral corners and are not rectangular. But, the frustums are also “rectangular” according to SP22. The frustum is a generalized-cylinder with a straight axis and with two end-sections that are normal to the axis. SP22 (p. 11) referred to this property of the end-sections as being rectangular to discuss the third illusion but not the first illusion.

The second illusion is an impossible pair of objects (Figure 10 in SP22). One of the objects is a planar ring. The other object is a composition of square-cylinders and apparently-square-cylinders that are not actually square-cylinders. These two objects partially occlude one another in a way that looks impossible. SP22 claimed that this illusion was caused by the apparent-rectangularity of the cylinders. Note that the perceived rectangular shapes of the cylinders are mirror-symmetrical and compact but their actual shapes are asymmetrical and less compact. So, the mirror-symmetry and the compactness of the cylinders can also explain this illusion (Sawada, 2010; Pizlo et al., 2014, see Figure 1E).

The third illusion is Sugihara (2016) “Right-Facing Arrow.” The original illusion uses a cylinder with an open-top. The boundary-contour of the top, which is non-planar, is perceived as planar. The plane of this apparently-planar contour is normal to the axis of the cylinder. This property of the contour is also referred to as being rectangular by SP22 (p. 11). This illusion could be reproduced without this rectangularity (Figure 1F).

The third illusion can be explained by two types of degenerate views and 2D mirror-symmetry. Some sharp-angles of the contour are not visible in the original view and a sharp-angle in the view is a projection of a smoothly-curved segment of the contour (Figure 1F). The entire contour appears to be 2D mirror-symmetrical in the original view (Figure 1F) and in another view (Figure 1G). So, some other views that are close to these views are approximately 2D skew-mirror-symmetrical.

The contour of the illusion is 3D rotation-symmetrical and it is hard to see this symmetry. The visual system processes 3D rotational-symmetry and 3D mirror-symmetry quite differently (Sawada and Zaidi, 2018; Sawada and Farshchi, 2022). Perception of these two types of 3D symmetry should be discussed separately.

## Author contributions

TS and MD contributed to the conception, analysis, and discussion. TS wrote the first draft of the manuscript. Both authors reviewed the manuscript and approved the final version of the manuscript for submission.

## Footnotes

^1^For example, the degree of non-rectangularity of a trihedral corner can be defined as the sum of the differences of the angles forming the corner from 90°.
